# The value of echocardiography in the staging of preexcitation syndrome and the assessment of left ventricular wall dyskinesia in children

**DOI:** 10.3389/fped.2025.1567172

**Published:** 2025-04-25

**Authors:** Yahui Yuan, Shu Li, Jun Chen, Yu Mao, Ming Yang, Shiwei Yang, Wentao Kong, Hao Liu

**Affiliations:** ^1^ Department of Ultrasound, Children’s Hospital of Nanjing Medical University, Nanjing, China; ^2^ Department of Radiology, Children’s Hospital of Nanjing Medical University, Nanjing, China; ^3^ Department of Cardiology, Children’s Hospital of Nanjing Medical University, Nanjing, China; ^4^ Department of Ultrasound, Nanjing Drum Tower Hospital of Nanjing University Medical School, Nanjing, China

**Keywords:** preexcitation syndrome, echocardiography, preexcitation cardiomyopathy, two-dimensional speckle tracking imaging, children, wolff-Parkinson-White syndrome

## Abstract

**Background:**

The incidence of Wolff-Parkinson-White syndrome (WPWs) in the pediatric population is increasing recently. Conventional echocardiography lacks specificity and was limited to assessing the effects of WPWs on cardiac structure, while changes in cardiac function, ventricular wall dysfunction and different subtypes of WPWs were less commonly addressed. Whether WPWs causes cardiac decompensation and ventricular dyskinesia was controversial. Whether echocardiographic multiparameter indices can improve the diagnostic specificity and guide the classification of WPWs and assess the recovery of left ventricular (LV) synchrony and ventricular wall dyskinesia in patients after radiofrequency ablation (RFA) is a very important direction of research.

**Objectives:**

To analyse the echocardiographic performance of patients with WPWs: (1) to assess the hazard of WPWs on cardiac function and LV wall dyskinesia using ultrasound multiparameters, with the aim of exploring in depth the pattern of effect of WPWs on cardiac function and motion, (2) to attempt to use echocardiography for staging WPWs, and (3) to predict patient recovery after RFA.

**Methods:**

The clinical, echocardiographic and RFA data of 75 children with RFA-confirmed WPWs from January 2019 to December 2024 were retrospectively analysed and compared with 60 healthy controls during the same period. All statistical analyses were carried out using SPSS 26.0 and *P*-values <0.05 were considered statistically significant.

**Results:**

Two-dimensional echocardiography demonstrated significant LV enlargement, reduced LV systolic function, and significant ventricular wall dyskinesia in 14 of 75 patients, basal segmental septal dyskinesia in 5, and LV posterior wall dyskinesia in 4. The remaining 52 patients did not have significant ventricular wall dyskinesia. Multiparameters showed that WPWs patients compared with healthy controls: GLS (−18.16 ± 3.54% vs. −22.88 ± 0.71%), TD_SD_ (38.88 ± 6.77 ms vs. 24.03 ± 1.90 ms), TD_RV−LV_ (41.18 ± 7.21 ms vs. 24.32 ± 1.94 ms), PSD (35.26 ± 5.42 ms vs. 22.44 ± 2.23 ms), and MPTD (196.92 ± 61.41 ms vs. 100.55 ± 10.25 ms) were all statistically different from each other (*P* < 0.05). LVEDD *Z* score (1.07 ± 0.84 vs. 1.05 ± 0.56), LVEF (61.20 ± 9.02% vs. 66.52 ± 3.16%) was not significantly different between the two groups (*P* > 0.05). Seventy-five patients underwent RFA among them, 28 cases of type A bypass and 47 cases of type B bypass, analysed the longitudinal strain bull's-eye diagram of the LV, it was concluded that the GLS of the inferior, inferior lateral and anterior lateral walls of type A bypass was significantly reduced, and the GLS of the anterior wall, anterior septum and inferior septum of type B bypass was significantly reduced, with the most significant in the basal segment, followed by the intermediate segment, and the apical region was not involved. All the postoperative ultrasound parameters were better than the preoperative ones, and the results of the 3-month postoperative review showed that there was a difference between the ventricular synchronisation indexes and those of the healthy group, suggesting that the LV synchronisation had not yet completely returned to normal. ROC curve analysis showed GLS, TD_SD_, TD_RV−LV_, PSD and MPTD could predict the prognosis of recovering post RFA.

**Conclusions:**

Conventional echocardiography and two-dimensional speckle tracking imaging have the capacity to provide reference data for the reduction of cardiac function and ventricular wall motion disorder caused by WPWs. Furthermore, the longitudinal strain bull's eye map of two-dimensional speckle tracking imaging has the potential to guide the classification of WPWs. Furthermore, a multitude of echocardiographic parameters have been shown to predict the prognosis of recovering post RFA.

## 1 Introduction

WPWs is a common cardiac arrhythmia with a prevalence of about 0.1%–0.3% (1, 2), and it is the most common cause of cardiac dyskinesia in children (3). Long-term asynchronous cardiac motion can lead to cardiomyocyte degeneration and ventricular structural remodelling, and in severe cases, LV dilatation and systolic function reduction in severe cases. Some researchers referred to this condition as preexcited cardiomyopathy or ventricular preexcited dilated cardiomyopathy (4). WPWs is mainly diagnosed by surface ECG, which has limited sensitivity and cannot evaluate myocardial motion and function. Echocardiography commonly used in clinical practice, provides multi-parameter assessments of heart structure, motion, function and synchronisation of the heart (5). Currently, echocardiography serves as the primary tool for assessing the impact of WPWs on cardiac function (6–8). A few literatures reported (9–11) that echocardiography can be used to evaluate the effect of WPWs on the ventricular wall and help in the localization of ectopic targets. However, existing studies have yet to reach a consensus regarding the correlation between echocardiographic parameters and WPWs, necessitating further clinical investigation. This study intends to assess the LV wall dyskinesia in children with WPWs by conventional echocardiography and 2D speckle tracking imaging (2D-STI) multiparameters and tries to analyse the changes of these parameters in its different subtypes, aiming to improve the in-depth understanding of the disease and further evaluate the recovery of cardiac structure and motion after RFA, providing an objective basis for the prognosis of the clinical treatment of the disease.

## 2 Methods

### 2.1 Subject of the study

This study was approved by the institutional review boards of Children's Hospital of Nanjing Medical University (202309113-1). The requirement for informed consent was waived because of the retrospective study design.

Seventy-five children with WPWs confirmed by RFA electrophysiology were retrospectively collected from January 2019 to December 2024 in the Children's Hospital of Nanjing Medical University. Inclusion criteria: (1) body surface electrocardiogram results met the diagnostic criteria of WPW (12): manifested as shortening of P-R interval, widening of QRS wave cluster with pre-excitation wave, (2) unresponsive to medical therapy or frequent supraventricular tachycardia were hospitalized for RFA, (3) clear echocardiographic sonogram. Exclusion criteria: (1) congenital heart disease; (2) cardiomyopathy, atrial fibrillation, sustained supraventricular tachycardia, and split-frequency interventricular preexcitation. All children were required to stop antiarrhythmic drugs for at least 5 half-lives before RFA. Periodic 24-h ambulatory electrocardiograms were performed on each patient to determine the presence of acute tachycardia episodes and the presence of supraventricular tachycardia lasting up to 12 h or tachycardia lasting up to 12 h.

Control group: 60 healthy children were selected as the control group. Inclusion criteria: (1) normal echocardiographic and electrocardiographic findings, (2) no obvious clinical symptoms. Exclusion criteria: (1) previous history of cardiac disease, (2) previous history of taking medication for cardiovascular disease.

The study group was divided into a type A bypass group (group A) and a type B bypass group (group B) according to the location of the bypass tracts in RFA (type A WPW atrial bypass was mainly located on the left side and type B WPW atrial bypass was mainly located on the right side).

### 2.2 Instruments and methods

The GE Vivid E95 ultrasound diagnostic instrument was used, and the M5SC-D phased array probe was selected to optimise the width of the swept sector and the image depth, and the frame rate was adjusted (>60 frames), and the electrocardiographic gating was connected, and the examined children were placed in the left lateral position, with the anterior thorax fully exposed, and examined when the heart rate was stable at a quiet state. Conventional echocardiography was used to take standard views and measurements; M-mode ultrasound was used to take standard left ventricular longitudinal views at the level of the notochord perpendicular to the interventricular septum; Tissue Doppler (TD) was used to take standard views of the LV in the apical 4-chamber, 2-chamber, and apical longitudinal views at the parietal level; TD was used to activate the coloured view of the LV and adjust the sector to encompass the entire LV, reduce the depth and sector width to focus on the LV, and adjust the overall colour. The overall colour gain was adjusted to clearly depict the myocardium, and the time-to-peak velocity curve was retained. 2-Dimensional Speckle Tracking Imaging (2D-STI) used the aortic spectrum to determine the aortic valve closure time, and the three standard views of the parasternal LV apical 4-chamber, 2-chamber, and apical long-axis were depicted. Three standard sections of LV endocardium were traced in the parasternal LV apical four-chamber heart, two-chamber heart, and apical long-axis, and longitudinal strain parameters of the LV myocardium were automatically calculated by the software (11). All patients were examined by the same attending cardiovascular ultrasound physician. Each echocardiographic parameter was calculated as the average of three cardiac cycles. To avoid subjective bias, the physician performing the echocardiogram was unaware of the patient's condition.

Conventional echocardiographic and M-mode echocardiographic parameters: (1) left ventricular end diastolic diameter *Z* score (LVEDD *Z*), use the app called Cardio *Z* which was designed, developed and managed by UBQO Limited; (2) left ventricular ejection fraction LVEF, obtained by Simpson's biplane method; (3) M-mode observation of interventricular septum and left ventricular posterior wall motion. The data obtained were corrected for body surface area.

Indicators of ventricular synchronisation: Tissu Doppler TD Intraventricular synchronisation indicator TD_SD_ and interventricular synchronisation indicator TD_RV−LV_: Time difference measured on the time-to-peak velocity curve.

2D-STI parameters: EchoPAC (Version201) workstation was used for offline image processing and analysis. Including: (1) LV myocardium longitudinal peak strain and strain peak time bull's-eye, (2) LV myocardium peak strain dispersion (PSD), (3) maximum peak time difference (MPTD), and (4) LV myocardium longitudinal strain (GLS). The LV segments were divided into 17 segments according to the guidelines (13).

### 2.3 Statistical methods

Data were analysed using SPSS 26.0 statistical software. Measurements that conformed to normal distribution were expressed as mean ± standard deviation, and *t*-tests were used to compare the parameters between the control group and the case group and to compare the ultrasound parameters between the A-type bypass group, the B-type bypass group and the normal control group. Comparison of ultrasound measurements between groups at different time points was performed by one-way ANOVA; count data were expressed as cases (percentage). Comparison of count data was performed using the chi-square test. The parameters with statistical significance in the univariate analysis were incorporated into the binary logistic regression model to explore the independent influencing factors of recovering post RFA and the ROC curve was plotted and the area under the curve AUC was calculated for statistical analysis. *P* < 0.05 was considered statistically significant.

## 3 Results

### 3.1 General information

There were 75 patients in the WPWs group, 43 males and 32 females, aged 4–14 years, with an average of (8.5 ± 3.3) years old, and 60 patients in the control group, 35 males and 25 females, aged 4–15 years, with an average of (8.7 ± 2.7) years old. There was no significant difference in age and gender between the two groups (*P* > 0.05). Group A 28 cases [16 males, 12 females, age 6–12 years, average (9.2 ± 2.5) years], group B 47 cases [29 males, 18 females, age 7–14 years, average (9.8 ± 2.8) years], the difference between the two groups in terms of age and gender is not statistically significant (*P* > 0.05). Seventy-five cases of radiofrequency ablation were treated with accurate positioning of the ectopic target point in the operation and were ablated successfully, and the electrocardiograms were restored to normal. The electrocardiogram was restored to normal.

### 3.2 Routine echocardiographic performance in patients with WPWs

**Figure 1:**
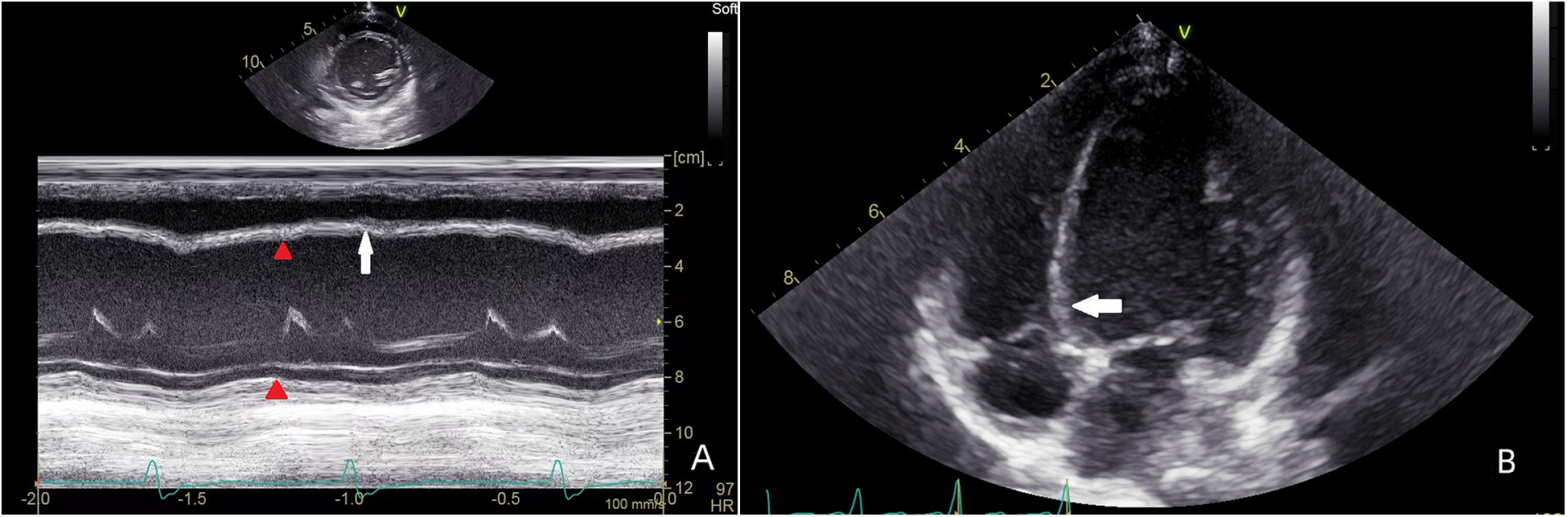
Patient, male, 7 years old, with preexcitation syndrome on electrocardiogram, **(A)** preoperative M-mode echocardiogram shows the septum in congruent motion with the posterior wall of the LV (red triangle), with a localised convexity of the septum towards the right ventricle (white arrow); **(B)** echocardiogram of the parietal four-chambered heart shows an enlarged LV, with a bias of the septum towards the right ventricle, and localised dilatation of the basal segments (white arrow) in a paradoxical motion with the septum as a whole.

**Figure 2:**
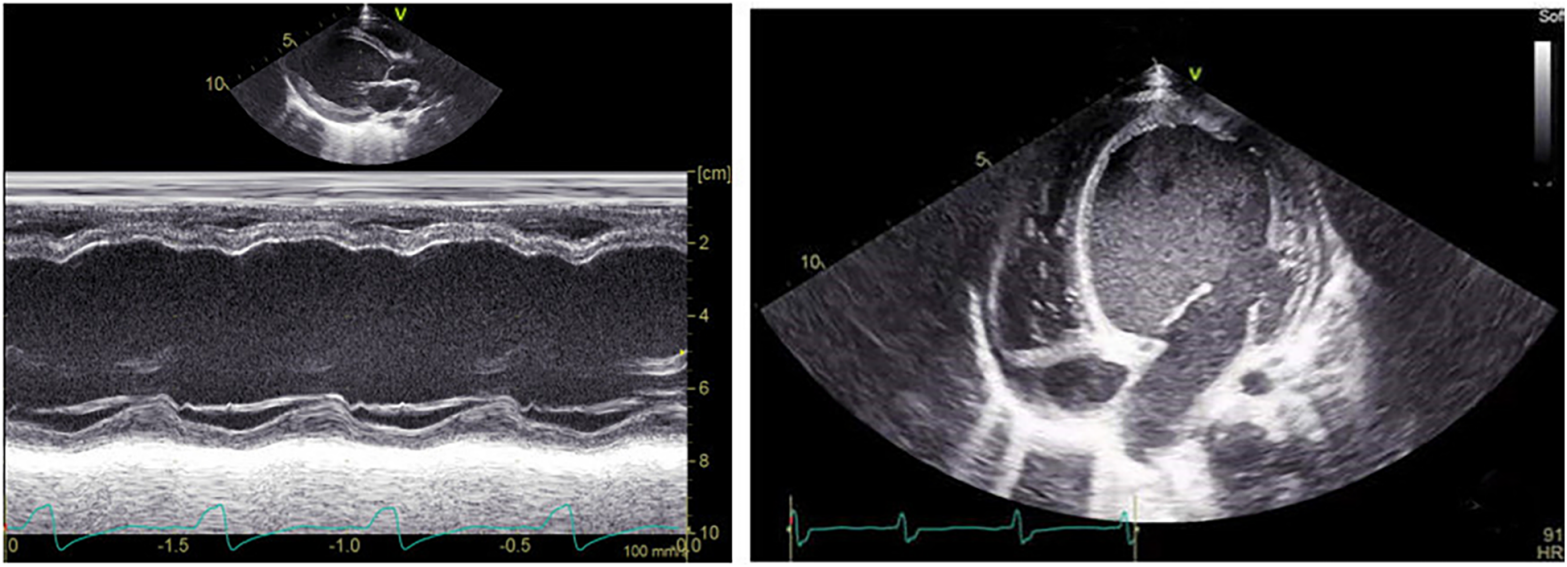
Patient, male, 11 years old, with preexcited dilated cardiomyopathy, left M-mode echocardiogram shows decreased septal and posterior left ventricular wall motion and reduced ejection fraction; transthoracic apical 4-chamber view shows spherical enlargement of the LV and thinning of the ventricular wall.

LV enlargement was seen in 45 of the 75 patients (LVEDD 42.7 ± 4.4 mm, *Z* score 1.89 ± 0.21), of which 14 had preexcited dilated cardiomyopathy, which was manifested by marked enlargement of the LV cavity (LVEDD 48.2 ± 7.3 mm, *Z* score 2.33 ± 0.37), reduced LV systolic function (LVEF 38.5 ± 8.9%), and markedly impaired ventricular wall motion, and in 5 patients, basal segment of the ventricular septum appeared in the M-mode echocardiogram. There was no significant ventricular wall motion disorder on conventional echocardiography in the remaining 30 cases; they had normal LV size (LVEDD 36.5 ± 5.5 mm, *Z* score 0.89 ± 0.14), 4 cases showed incoordination of the posterior wall of the LV on M-mode echocardiography, and 26 cases had no significant abnormality on conventional echocardiography. A total of 23 patients 2D echocardiograms showed incongruous ventricular wall motion, including 19 cases of B-mode, with thinning of the basal segment during systole and paradoxical motion (Figure 1). Fourteen cases fulfilled the diagnostic criteria for dilated cardiomyopathy, and cardiomyopathy and other causes were excluded on the basis of the chief complaint, the frequency of supraventricular tachycardia, and the periodic 24-h ambulatory electrocardiograms. The children presented with the clinical signs and symptoms of chronic congestive heart failure, and the LV presented with a spherical enlargement, and reduced LV ejection fraction on M-mode echocardiography (Figure 2); in four cases of type A, the posterior wall of the LV showed movement in the same direction as the interventricular septum on M-mode echocardiography.

### 3.3 Comparison of ultrasound multiparameters between WPWs group and healthy control group

Conventional echocardiographic parameters: LVEDD *Z* (1.07 ± 0.84 vs. 1.05 ± 0.56, t = 1.422, *P* = 0.06); LVEF (61.20 ± 9.02% vs. 66.52 ± 3.16%, t = 0.824, *P* = 0.13). TD_SD_ (38.88 ± 5.79 ms vs. 24.03 ± 14.85 ms, t = 16.232, *P* < 0.01); TD_RV−LV_ (41.18 ± 7.21 ms vs. 24.32 ± 1.94 ms, t = 15.331, *P* < 0.01). 2D-STI parameters: GLS (−18.16 ± 3.54% vs. −22.88 ± 0.71%, t = 12.021, *P* < 0.01); PSD (35.26 ± 5.42 ms vs. 22.44 ± 2.23 ms, t = 7.832, *P* < 0.01); MPTD (196.92 ± 61.41 ms vs. 100.55 ± 10.25 ms, t = 8.322, *P* < 0.01).

### 3.4 Ultrasound parameters between WPWs subtypes

**Figure 3:**
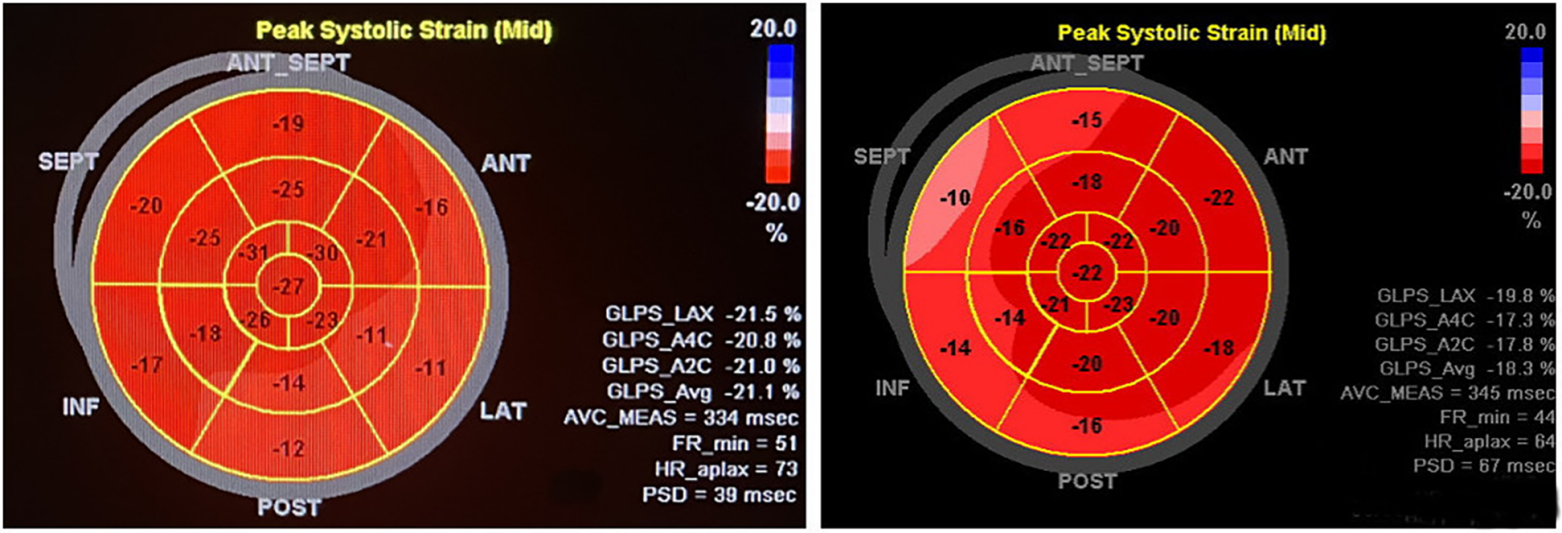
The patient on the left, male, 11 years old, was admitted to the hospital for radiofrequency ablation for pre-excitation syndrome, with intraoperative localisation of the bypass as the left bypass, type A. Preoperative two-dimensional speckle-tracking imaging of longitudinal myocardial strain bull's-eye showed lower-than-normal strain values in the inferior wall of the LV (−12%), inferior lateral wall (−11%), and anterolateral wall (−16%), notably in the basal segments. The patient on the right, female, 10 years old, was admitted to the hospital for radiofrequency ablation with confirmed diagnosis of preexcitation cardiomyopathy, with intraoperative electrophysiological clarification of the right bypass, Type B. Preoperative two-dimensional speckle tracking imaging of longitudinal strain in the left ventricular myocardium bull's-eye view shows lower than normal strain values in the basal and middle segments of the anterior wall (−15% and −18%), anterior septum (−10% and −16%), inferior septum (−14% and −14%), and inferior wall (−16% and −20%).

There was no difference in LVEDD *Z* and LVEF in group A patients compared with the control group (*P* *>* *0.05*), and there were differences in TD_SD_, TD_RV−LV,_ GLS, PSD, and MPTD (*P* *<* *0.05*); all ultrasound parameters differed in group B patients compared with the control group (*P* < 0.05); and there were differences in each ultrasound parameter between type A and type B children (*P* < 0.05) (Table 1). 2D-STI of longitudinal peak strain in various segments of the LV bull's-eye analysis of longitudinal strain values of the basal segment of the anterior wall, the inferior septum, the inferior wall, the inferior lateral wall, and the basal and intermediate segments of the anterior lateral wall of patients in group A were statistically different from those of the control group (*P* < 0.05), and the lower wall and the inferior lateral wall had more pronounced reduction of the GLS; in patients of group B the anterior wall, the anterior septum, the inferior septum and the basal and intermediate segments of the inferior wall, the inferior lateral wall and the anterior lateral wall The longitudinal strain values of the basal segment were statistically different from those of the control group (*P* < 0.05), and the GLS reduction was more obvious in the anterior wall, anterior septum and inferior septum. There was no statistically significant difference in the longitudinal strain values of the apical segments between the two groups and the control group. (Table 2 and Figure 3).

**Table 1:** Comparison of ultrasound parameters between type A bypass and type B bypass groups and normal control group (x ± s).

Ultrasonic parameters	Group A (*n* = 28)	Group B (*n* = 47)	Healthy control group (*n* = 60)	95% CI	*F*	*P*
LVEDD Z	1.09 ± 0.09^c^	1.51 ± 0.08^a^,^b^	1.05 ± 0.56^c^	0.853–1.243	17.183	0.006
LVEF(%)	62.3 ± 1.7^c^	54.2 ± 10.2^a^,^b^	66.52 ± 3.16^c^	56.332–64.1536	39.113	<0.001
GLS(%)	−20.1 ± 0.7^a^,^c^	−18.2 ± 2.4^a^,^b^	−22.88 ± 0.71^b^,^c^	−16.175 to −22.321	272.798	<0.001
TD_SD_	35.2 ± 7.3^a^,^c^	43.5 ± 10.2^a^,^b^	24.03 ± 1.90^b^,^c^	22.124–41.332	75.236	<0.001
TD_RV−LV_	37.6 ± 5.9^a^,^c^	42.5 ± 6.6^a^,^b^	24.32 ± 1.94^b^,^c^	23.146–41.726	69.846	<0.001
PSD	28.4 ± 3.5^a^,^c^	44.6 ± 16.3^a^,^b^	22.44 ± 2.23^b^,^c^	21.877–35.476	65.091	<0.001
MPTD	139.5 ± 20.3^a^,^c^	228.2 ± 95.8^a^,^b^	100.55 ± 10.25^b^,^c^	99.116–220.176	88.706	<0.001

^a^ *P* < 0.05 vs. control; ^b^*P* < 0.05 vs. type A bypass group; ^c^*P* < 0.05 vs. type B bypass group. LVEDD Z: Left ventricular end-diastolic internal diameter *Z* Score; LVEF, left ventricular ejection fraction; LV, myocardial peak strain dispersion by segment; MPTD, maximum time to peak difference; GLS, global longitudinal strain of the left ventricular myocardium; PSD, Peak strain dispersion of left ventricular myocardium in each segment; TD_SD_, longitudinal tissue Doppler rate difference between left and right ventricles. Doppler rate difference; TD_RV−LV_, longitudinal tissue Doppler rate difference between the left and right ventricles.

**Table 2:** Comparison of longitudinal peak strain in various segments of the LV wall in WPWs patients, groups A, B and controls (x ± s) (%).

LV segment		Group A (*n* = 28)	Group B (*n* = 47)	Healthy control group (*n* = 60)
Basal segment	Front wall	−16.2 ± 4.7#	−13.6 ± 5.3#	−20.3 ± 3.7
	Front interval	−18.9 ± 5.2	−11.2 ± 5.1#	−20.5 ± 4.1
	Lower interval	−16.8 ± 5.2#	−14.2 ± 4.6#	−20.3 ± 3.9
	Lower wall	−13.3 ± 4.5#	−15.4 ± 4.1#	−20.6 ± 3.7
	Lower sidewall	−10.9 ± 4.5#	−18.0 ± 4.1#	−20.8 ± 4.0
	Front side wall	−15.4 ± 4.6#	−19.9 ± 4.2	−20.3 ± 4.1
Middle section	Front wall	−18.8 ± 4.2	−17.5 ± 4.1#	−21.2 ± 3.3
	Front interval	−19.2 ± 4.6	−15.1 ± 4.5#	−20.7 ± 6.1
	Lower interval	−17.8 ± 4.3#	−15.5 ± 4.7#	−21.2 ± 3.1
	Lower wall	−15.7 ± 4.6#	−18.1 ± 4.3#	−20.9 ± 3.5
	Lower sidewall	−12.1 ± 4.2#	−18.9 ± 5.2	−20.7 ± 3.7
	Front side wall	−18.4 ± 4.5#	−20.1 ± 4.4	−21.2 ± 3.9
Apical segment	Front wall	−19.2 ± 4.2	−19.9 ± 3.9	−20.0 ± 3.6
	Front interval	−20.8 ± 4.1	−21.0 ± 3.6	−21.2 ± 3.9
	Lower interval	−20.8 ± 4.1	−19.8 ± 4.3	−21.2 ± 3.5
	Lower wall	−19.9 ± 4.7	−20.7 ± 4.2	−21.1 ± 3.9
	Lower sidewall	−19.8 ± 4.5	−20.5 ± 4.7	−21.2 ± 3.9
	Front side wall	−19.7 ± 4.6	−20.3 ± 4.4	−21.2 ± 3.2
Apex		−23.5 ± 4.7	−24.1 ± 4.2	−25.6 ± 5.1

^#^ WPW patients preoperatively compared to controls, *P* < 0.05.

### 3.5 Comparison of ultrasound parameters in the study group before and after RFA and the control group

**Figure 4:**
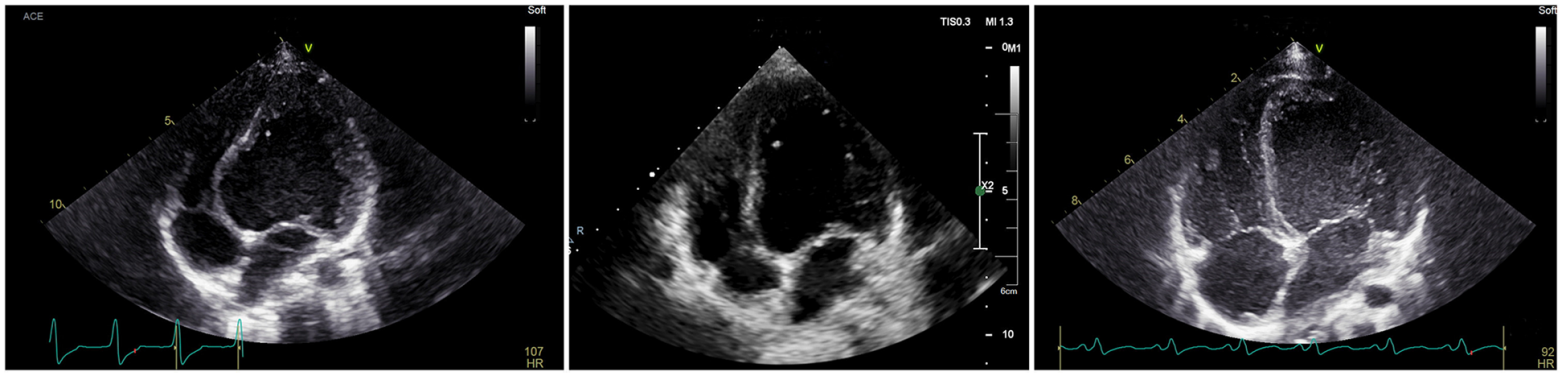
The patient, male, 8 years old, was admitted to the hospital for RFA treatment for WPWs, and intraoperative diagnosis of type B bypass was made. Preoperatively, the LV of the parasternal four-chambered heart was globularly dilated, and the septum was significantly convex towards the right ventricle; on review 1 week after the operation, the LV was significantly smaller than before the operation, and the base of the septum was slightly convex towards the right ventricle; on review 3 month after the operation, the four-chambered heart showed that the left and right hearts were in good proportion to each other.

**Figure 5:**
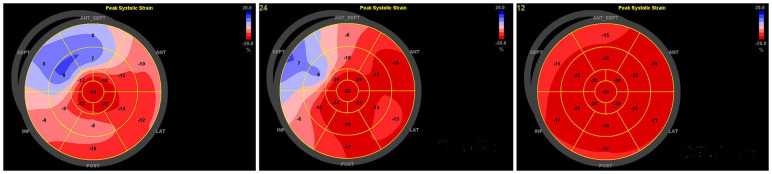
Patient, female, 11 years old, intraoperative diagnosis of type B bypass, preoperative-1 week postoperative-3 month postoperative longitudinal strain bull's-eye view of the left ventricular myocardium showed a gradual increase in the values of anterior, anterior septal, inferior septal, and inferior wall strains, and the results of the 1-month review had returned to almost normal.

**Figure 6:**
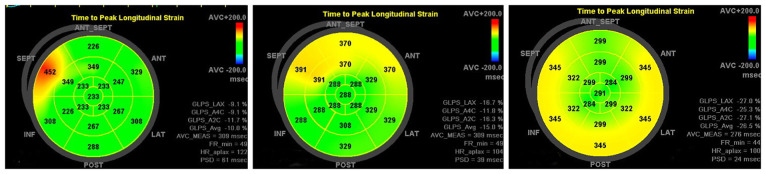
Patient, male, 10 years old, intraoperative diagnosis of type B bypass, preoperative-1 week postoperative-3 month postoperative peak time strain bull's-eye diagram showing PSD decreasing from 61 ms to 39 ms and finally 24 ms, returning to normal values.

Table 3 shows that the preoperative ultrasound parameters of the RFA group deviated from the normal control group, and the difference between the groups was statistically significant (*P* < 0.001); in the 1-week postoperative review, the abnormalities of the ventricular wall motion disappeared, and the parameters were better than the preoperative parameters (Figures 4–6), and the difference was statistically significant (*P* < 0.001), among which, the LVEDD *Z* and LVEF did not differ from that of the control group (*P* > 0.05), and the remaining parameters were different from the control group (*P* < 0.001); the difference between LVEDD *Z*, LVEF and GLS in the 3-month postoperative group and the control group was not statistically significant (*P* > 0.05), and the parameters of ventricular synchronisation were still different from the control group (*P* < 0.001).

**Figure 7:**
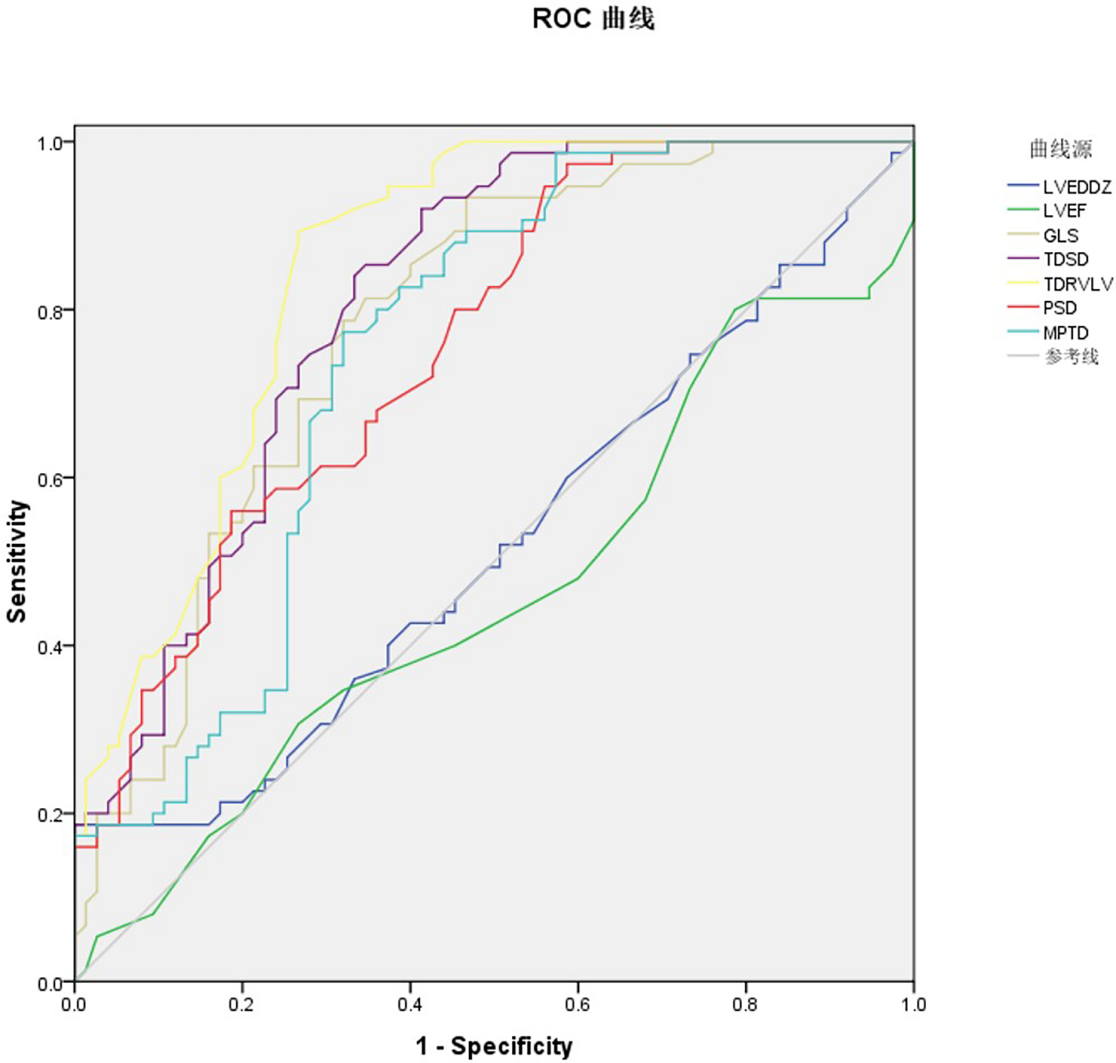
ROC curve of ultrasound multi-parameters predicting recovering post RFA prognosis in WPW patients.

**Table 3:** Comparison of ultrasound parameters between patients in the study group preoperatively, 1 week postoperatively, and 3 month postoperatively with the control group (x ± s).

Ultrasonic parameters	Preoperative group (*n* = 75)	1 week postoperative group (*n* = 75)	3 month postoperative group (*n* = 75)	Healthy control group (*n* = 60)	95% CI	F	*P*
LVEDD Z	1.07 ± 0.84	0.95 ± 0.63	0.83 ± 0.53	1.05 ± 0.56	0.877–1.265	6.142	<0.001
LVEF(%)	61.20 ± 9.02	63.12 ± 5.51	65.71 ± 3.19	66.52 ± 3.16	59.124–63.276	34.212	<0.001
GLS(%)	−18.16 ± 3.54^a^^,^^b^^,^^c^	−21.09 ± 1.41^a^^,^^b^^,^^d^	−22.39 ± 0.71^c^^,^^d^	−22.88 ± 0.71^c^^,^^d^	−17.374 to −22.972	319.708	<0.001
TD_SD_(ms)	38.88 ± 6.77^a^^,^^b^^,^^c^	33.09 ± 5.23^a^^,^^b^^,^^d^	27.60 ± 3.80^a^^,^^c^^,^^d^	24.03 ± 1.90^c^^,^^d^	23.789–40.437	415.840	<0.001
TD_RV−LV_ (ms)	41.18 ± 7.21^a^^,^^c^	34.35 ± 5.00^a^^,^^b^	28.06 ± 3.59^a^^,^^c^^,^^d^	24.32 ± 1.94^b^^,^^c^^,^^d^	24.075–42.843	143.889	<0.001
PSD (ms)	35.26 ± 5.42^a^^,^^b^^,^^c^	30.15 ± 4.59^a^^,^^b^^,^^d^	25.77 ± 3.71^a^^,^^c^^,^^d^	22.44 ± 2.23^b^^,^^d^	22.154–36.510	282.516	<0.001
MPTD (ms)	196.92 ± 61.41^a^^,^^c^	157.52 ± 47.881^a^^,^^b^	113.81 ± 27.14^a^^,^^b^^,^^d^	100.55 ± 10.25^c^^,^^d^	99.243–211.049	201.122	<0.001

^a^ *P* < 0.05 vs. control group; ^b^*P* < 0.05 vs. 3-month postoperative group; ^c^*P* < 0.05 vs. 1-week postoperative group; ^d^*P* < 0.05 vs. preoperative group. LVEDD *Z*, Left ventricular end-diastolic internal diameter *Z* score; LVEF, Left ventricular ejection fraction; PSD, Peak strain dispersion of left ventricular myocardium in each segment; MPTD, Maximum Peak Tolerance Time Difference; GLS, Overall longitudinal strain of the left ventricular myocardium; TD_SD_, Longitudinal tissue Doppler rate difference within the ventricle; TD_RV−LV_, Longitudinal tissue Doppler rate difference between the left and right ventricles.

### 3.6 Predicting the prognosis of recovering post RFA using ultrasound multi-parameters

The receiver operating characteristic (ROC) curves were drawn respectively for the seven ultrasound multi-parameter. The AUC for LVEDD *Z* was 0.519, 95% CI = 0.462–0.612, *P* = 0.031 with the cutoff value 1.025 the sensitivity 40.0% and the specificity 62.7%. The AUC for LVEF was 0.462, 95% CI = 0.369–0.555, *P* = 0.121 with the cutoff value 66.5% the sensitivity 30.7% and the specificity 73.3%. LVEDD *Z* and LVEF of conventional echocardiography parameters have no statistical significance in predicting the prognosis of recovering post RFA. The AUC for GLS was 0.782, 95% CI = 0.708–0.856, *P* < 0.05 with the cutoff value −20.450 the sensitivity 89.3% and the specificity 73.4%. The AUC for TD_SD_ was 0.809, 95% CI = 0.740–0.878, *P* < 0.05 with the cutoff value −31.750 the sensitivity 93.3% and the specificity 53.3%. The AUC for TD_RV−LV_ was 0.849, 95% CI = 0.787–0.911, *P* < 0.05 with the cutoff value 33.800, the sensitivity 92.0% and the specificity 57.3%. The AUC for PSD was 0.757, 95% CI = 0.681–0.832, *P* < 0.05 with the cutoff value 30.650, the sensitivity 80.0% and the specificity 54.7%. The AUC for MPTD was 0.747, 95% CI = 0.667–0.827, *P* < 0.05 with the cutoff value 141.150, the sensitivity 84.0% and the specificity 58.7%. The above five indicators can predict the prognosis of recovering post RFA (Figure 7, Table 4).

**Table 4:** Predictive accuracy of ultrasound variables for recovering post RAF prognosis.

Ultrasonic parameters	AUC	95% CI	Cutoff value	Sensitivity (%)	Specificity (%)
LVEDD Z	0.519	0.462–0.612	1.025	40.0	62.7
LVEF(%)	0.462	0.369–0.555	66.5	30.7	73.3
GLS(%)	0.782	0.708–0.856	−20.450	89.3	73.4
TD_SD_	0.809	0.740–0.878	31.750	93.3	53.3
TD_RV−LV_	0.849	0.787–0.911	33.800	92.0	57.3
PSD	0.757	0.681–0.832	30.650	80.0	54.7
MPTD	0.747	0.667–0.827	141.150	84.0	58.7

LVEDD *Z*, Left ventricular end-diastolic internal diameter *Z* Score; LVEF, Left ventricular ejection fraction; PSD, Peak strain dispersion of left ventricular myocardium in each segment; MPTD, Maximum Peak Tolerance Time Difference; GLS, Overall longitudinal strain of the left ventricular myocardium; TD_SD_, Longitudinal tissue Doppler rate difference within the ventricle; TD_RV−LV_, Longitudinal tissue Doppler rate difference between the left and right ventricles.

## 4 Discussion

In normal physiology, the atrioventricular node is the only electrical conduction structure between the atria and the ventricles. When there is an abnormal bypass exists, the impulses from the sinus node travel not only through the normal conduction pathway, but also through this faster bypass, causing premature myocardial activation and arrhythmia, known as ventricular preexcitation (14). Common symptoms include episodic tachycardia, precordial discomfort, pain, palpitations and other related symptoms. Medical treatment of WPWs is usually ineffective, and RFA is a class Ⅰ indication for WPWs (15). After RFA to eliminate the abnormal pathway, cardiac conduction returns to normal and clinical symptoms usually resolve (16).

Currently, the diagnosis of WPWs mainly relies on the surface ECG, which shows ventricular excitation dyssynchrony due to the presence of the bypass channel. This manifests as shortened P-R intervals and widened QRS complexes with preexcitation waves, but its diagnostic efficacy is limited (12). Echocardiography offers high specificity for WPWs diagnosis, mainly focusing on the structural and motion changes like ventricular preexcitation dilated cardiomyopathy, characterized by LV, diminished and contradictory motion (11). In this study, 60% (45/75) of patients showed LV enlargement, and 30.7% (23/75) had discordant ventricular wall motion. The specificity of 2D echocardiography in the diagnosis of WPWs is relatively limited. 2D-STI and tissue doppler showed significant differences in myocardial function indices compared with healthy controls. The application of 2D-STI to the study of myocardial function has matured, and this technological index is more sensitive in responding to the myocardial work done (17). The longitudinal strain bull's-eye view of the LV revealed that almost all patients had localized wall motion reduction, with severe cases exhibiting contradictory local myocardial motion, whereas 2D echocardiography revealed wall motion abnormalities in only 23 cases. Further analysis of the longitudinal strain curve of the ventricular septum revealed two contraction peaks: the earlier one was early contraction with a small peak, and rebound motion occurred when the aortic valve was closed, and then the peak of contraction occurred again with the motion of the posterior wall. The bull's-eye plots of the dispersion of the peak strains of each segment of the LV myocardium showed the time to reach the peak of the myocardium of each segment, and correspondingly to the bull's-eye plots of longitudinal strains, the myocardial segments with decreasing strain values had long time to peak, and the LV contractions were not synchronous with the peaks. Longer LV contraction dyssynchrony was demonstrated by differences in the peak times of each segment, further illustrating the pattern of WPWs myocardial agitation in terms of objective indicators (18). In this study, tissue doppler and 2D-STI showed that all WPWs patients had biventricular and overall LV motion incoordination, evidenced by either early right ventricular contraction and posterior LV contraction or asynchronous contraction of the LV septum with the posterior wall. This may be due to the fact that in patients with WPWs, the affected septum contradicts the LV motion, and when it is contracted, it projects to the right ventricle and participates in the right ventricular contraction, which results in a rapid increase in right ventricular pressure, and the pulmonary valve opens relatively earlier than in other patients, resulting in faster pressure elevation. The opening time of the pulmonary valve is relatively advanced, while the LV cannot get maximum filling, resulting in delayed opening of the aortic valve, and a vicious circle between abnormal ventricular wall motion and the synchrony of biventricular motion, accelerating the damage of myocardial function (19).

Among WPWs three types, type C is rarer and type B is more common than type A (11), and the results of the present study are consistent with this conclusion. The ectopic targets of type A bypass are mainly on the left side, located in the inferior wall, inferior lateral wall, and anterior lateral wall, whereas type B bypass are mainly on the right side, located in the anterior wall, anterior septum, and inferior septum (20). The results of the present study showed a similar pattern, and the decrease in the GLS indicated that the longitudinal contractile function of the affected segments was reduced. However, in addition to the reduced GLS of the above segments, the GLS of the basal segment of the anterior wall, the basal segment of the inferior septum and the intermediate segment of the inferior septum in patients with type A; and the GLS of the inferior wall, the basal segment of the inferior lateral wall and the intermediate segment of the inferior wall in patients with type B differed from that of the normal control group, which indicated that the range of the affected segments was broader. The descending GLS segment shown in the bull's eye plot did not correspond exactly to the ectopic target, indicating that the specificity of classification based on GLS alone was insufficient. The GLS reduced segments of type A and B still overlapped with the location of the ectopic target, centred on the largest reduction in GLS segments and fluctuating to the surrounding parts of the segments, which caused their diminished motion. The different myocardial conduction locations of the ectopic targets are related to the myocardial regions that are excited in advance. When the clinical diagnosis of WPWs was made, a preliminary judgement of WPWs subtyping can be made based on the main involved segments shown in the 2D-STI bull's-eye diagram. According to the bull's eye map derived from 2D-STI revealed that the segment exhibiting the most significant reduction was selected as the center. This selection was consistent with the segment corresponding to the ectopic target, indicating that 2D-STI results may potentially provide objective data to localize the accessory pathway for classification of WPWs and provide a valuable clinical reference.

The results of this study showed that a minority of patients with type B bypass progressed to preexcitation cardiomyopathy, 14 patients showed dilated cardiomyopathy manifestations such as left ventricular enlargement and decreased LVEF. Because the ectopic target of type B accessory pathways is located on the right side, these pathways are closer to the right ventricle and ventricular septum compared to type A left accessory pathways. Consequently, excitation can propagate to the ventricular electrical pathway earlier, leading to premature excitation of associated myocardium and inducing contraction, which ultimately results in global myocardial dyscoordination. In contrast, the left accessory pathway is situated near the posterior wall of the LV, where excitation propagates later, thereby producing myocardial dyssynchrony at a delayed time point. The study demonstrated that GLS decreased more significantly in type B bypass compared to type A bypass, and the ventricular synchrony index deviated from the normal control group. The type B bypass was a right atrial bypass, which prematurely agitated the septum which was originally agitated relatively early in the normal heart, making the entire Asymmetric excitation of the LV (21), early excitation of the ventricular wall site due to the contractile movement of the heart is not yet fully diastolic, the ventricular wall is subjected to reduced preload, the cardiac resistance to contraction is reduced, so the local ventricular wall work is reduced, and the long-term effects of this lead to a reduction in the supply of coronary arteries, the ventricular wall thinning, and the ventricular wall to withstand the stress of the wall to be reduced (19), the study group in the 19 patients with B-type WPWs patients have abnormal ventricular wall movement, as manifested by localized basal segments of the ventricular septum showed contradictory motion to the septum as a whole, and M-mode showed isotropic motion of the septum and the posterior wall of the LV. The superimposition of both abnormal electrical and mechanical conduction mediates LV dysfunction and remodeling, which further develops, resulting in paradoxical septal motion, thinning of the myocardium, inability of the LV to achieve maximal filling, and a decrease in myocardial contractility and work done, ultimately leading to LV enlargement and a decrease in LVEF, and, in the most severe cases, to the manifestation of dilated cardiomyopathy (21). Compared with primary dilated cardiomyopathy, the prognosis of this disease is better, and normal electrophysiology is restored by RFA of the abnormal paracardiac target, LV myocardial contraction is synchronized, and LV systolic function is fully restored (4, 20).

The 2013 EHRA and AEPC Expert Consensus on Transcatheter Radiofrequency Ablation of Arrhythmias in Children (15) states that: (1) RFA may be used in infants with drug-refractory or life-threatening tachycardia, (2) Class Iindication for radiofrequency ablation was ventricular preexcitation due to drug-refractory or intolerable ventricular preexcitation, (3) Class IIa indication for catheter ablation is frequent premature ventricular contractions with symptoms and body weight ≥15 kg. RFA is the class I indication for WPWs, most patients with WPWs can be treated with antiarrhythmic drugs on a regular basis, which can reduce the occurrence of paroxysmal supraventricular tachycardia to a certain extent, however, the restoration of left ventricular function and ventricular wall motion will only be satisfactory with successful RFA (11).

All 75 patients with WPWs enrolled in this study were indicated for RFA. Ectopic targets were repeatedly searched for and successfully ablated during the procedure. Electrocardiogram and echocardiography were repeated 1 week and 3 months after RFA. Improvement in several echocardiographic indicators was considered a good prognosis for RFA. In this study, 1 week after receiving the procedure, all the indexes of echocardiography were significantly improved compared with the preoperative period, LVEDD and LVEF had returned to normal level, and GLS had returned to normal level at 3 month after the procedure, and the indexes of synchronicity were slightly reduced compared with the normal controls. ROC analysis showed that GLS, PSD, MPTD, TD_SD_, and TD_RV−LV_ five demonstrated superior performance in predicting the prognosis of recovering post RFA. Among these indicators, GLS was identified as the most effective parameter, with an AUC value of 0.782 (95% CI = 0.708–0.856, *P* < 0.05), The cutoff value for GLS was −20.450, yielding a sensitivity of 89.3% and a specificity of 73.4%. While the other four indices exhibited high sensitivity, their specificity was relatively insufficient. Consequently, GLS may serve as a promising diagnostic tool for screening clinically suspected WPWS cases. The study confirms that the RFA can effectively treat WPWs, and the function of the LV and the motion of ventricular wall were restored to normal, so that the WPWs is an indication of RFA, and the prognosis is good. Therefore, WPWs is an indication for RFA and has a good prognosis, which was consistent with the study by Uhm et al. (22). Echocardiography is a good way to monitor the postoperative effect of RFA and provides objective clinical data for the prognosis of patients with WPWs.

The shortcomings of this study: (1) the 2D-STI index was not comprehensive, failing to explore the myocardial damage more further in depth, and the comprehensiveness of the study will be emphasised in future follow-up studies; (2) there was no inclusion of C-type cases, which made the study of the disease incomplete; and (3) the time of review was not appropriately prolonged, and the long-term effect of the surgery was observed.

This study provides preliminary evidence that suggests the potential of echocardiographic multi-parameter analysis to serve as a reference for the assessment of left ventricular function and the monitoring of treatment in children diagnosed with WPWs. Furthermore, the study indicates that 2D-STI myocardial motion curve analysis can offer additional insights into the underlying principles of myocardial motion disorder in patients with WPWs. However, it is important to note that further research is necessary to enhance our understanding of the prognosis and predictive value of this condition. In the future, multi-center, prospective studies and long-term follow-up are needed to further verify the clinical relevance of these parameters.
